# Medical and Physical Intelligence

**Published:** 1800-10

**Authors:** 


### MEDICAL AND PHYSICAL INTELLIGENCE.

### *Obſervations on the Effects of Acetic Ether applied by Friction in Rheumatic Complaints, by* G. MARTIN. *Read at the Society of Medicine at Paris.*

WHEN I read the memoir of Citizon Sedillot the younger, on the acetic ether, inferted in the ſecond volume of the Recueil Periodique, &c. I was thinking what an uſeful remedy it would prove in our country, where the frequent change of weather often ſucceeding with aſtoniſhing rapidity, gives occaſion to many rheumatic complaints, if its efficacy was confirmed by numerous experiments carefully made. I ſoon found an opportunity of trying it, and the conſtant ſucceſs, together with the deſire of ſeeing it more generally employed by practitioners for diminiſhing the cruel pains of many people afflicted with rheumatiſms, has induced me to communicate to the Society of Medicine the following obſervations:

*Obſerv.* I. Citizen *Viard*, of a choleric temper, 55 years old, had for a long time been ſubject to rheumatic complaints, which attacked the ſuperior as well as inferior parts of the body, but had generally yielded to an antibilious treatment, and the topical applications that are commonly in uſe. The paroxyſms of theſe rheumatic complaints had not recurred ſince the time he began to uſe the hot baths of Rennes; having, however, omitted this for two years, he was, on the 13th of Prairial, attacked with an iſchiatic pain, extending from the hip to the extremity of the foot; the colour of the ſkin was not changed, but only a ſlight inflammation perceived on the lower and outer part of the leg. The two principal ſeats of the pain were about the hip and the articulation of the foot with the leg. Though the patient was without fever, he ſuffered extremely from the acute pains, which hardly let him keep the ſame ſituation for a moment; ſleep had almoſt left him, and he had no appetite, &c. After having uſed for nearly two months different remedies, externally and internally, that ſeemed to be indicated by the preſent ſymptoms, without obtaining much favourable change in the ſtate of the patient, who felt himſelf but very little relieved; I determined to employ the acetic ether in frictions, and got it accordingly prepared after the method of Cit. Sedillot. The patient was now rubbed on the whole extent of the pain every twelve hours, till ſix frictions had been applied, in which, each time, half an ounce of the ether was conſumed. After each friction, the patient was kept warm in his bed. On the third application, he began to feel himſelf relieved; and on the ſixth, he was ſo far cured, that he could walk the next day to to his country houfe, which was one league diftant from the town; whereas, three days before, he had not been able to move from one corner of the room to the other. After each friction an agreeable heat fpread itfelf over the fkin, which feemed to acquire more foftnefs, accompanied by a local perfpiration, foon becoming general, and continuing for fome time.

*Obferv.* 2. Cit. Efpallat was affected with a lumbago, attended with fuch violent pains, that from the firft attack he could not enjoy the leaft repofe. After having been treated, without avail, nearly a month with the ufual remedies, he was told by Cit. Viard of the fuccefsful effect he had himfelf experienced from the frictions with acetic ether. Being now applied to, I employed the ether accordingly in friction; at the the fourth application of which, the patient found himfelf confiderably relieved; and after the fixth, the cure was fo completely performed, that he left the bed and room, and was able to attend his ufual bufinefs. It is to be remarked, that each friction brought on likewife a pretty abundant perfpiration.

*Obferv.* 3. Cit. Touret was feized with ifchiatica, attended with moft violent pains. The patient having no fever, I adminiftered the ether in friction on all the painful parts; however, though the firft frictions produced an abundant local perfpiration, not any relief was procured to the patient, but the pains lafted with equal vehemence; notwithftanding this, the ether was continued, and at the fourteenth friction the patient felt himfelf a little better; the pains only remained at the hip, and the middle and outer part of the leg, and though they were ftill very excruciating, the remedy was continued. At the 24th friction the pains in the hip entirely difappeared, but in the leg a painful fenfation ftill exifted, though the patient was able to walk with facility, and appetite and reft were returned; the frictions are ftill continued with the hope of banifhing entirely the pain left in the leg. To thefe obfervations I could add many others, confirming the efficacy of that remedy. Some of my colleagues have likewife ufed it with fimilar fuccefs.

The above obfervations are an additional proof of what has been faid by Cit. Sedillot, of this remedy poffeffing the advantage of producing an agreeable heat on the fkin, and a very ufeful perfpiration, without augmenting the irritation or erethifm in thofe parts. It feems particularly to fatisfy the indication of opening the pores, and rendering the fkin permeable for the rheumatic matter.——*(Recueil Periodique de la Société de Medicine de Paris, No. xliii.)*

---

*Obfervations on the Ufe of Alkalis againft Accidents occafioned by Lightning.* By Cit. GAULTIER CLAUBRY.

THE effects of lightning are fometimes fo fingular, and vary fo much in every refpect, that it would be as tedious to undertake their hiftory as fuperfluous to attempt to explain them. The two following obfervations, however, will ftate fome facts which may perhaps perhaps feem extraordinary, and deferve attention; to one of which I have been witnefs, and of the other I was myfelf the fubject.

*Obferv.* 1. Chabot, a woman of the village of Groeft, near Blois, being occupied in preparing lixivium, was ftruck by a flafh of lightning, which extended from the left clavicle in a ftraight line to the inner ankle, and had caufed all this way a wound, two lines deep and about three lines wide, in which fhe at firft felt a flight degree of heat, increafing foon after fo as to give much pain. One of her acquaintance, anxious to affift her, applied the firft remedy which was neareft at hand, and poured upon her body a confiderable quantity of cold lixivium, which was made from the afhes of frefh wood. When I was called, I obferved in examining the wound, that all thofe places which had been touched by the lye were of a brownifh colour, and little fenfible; whereas thofe parts where it had not touched, had a high red colour, and were rifen in blifters, attended with violent heat and excruciating pains. Struck by this difference, I did not hefitate, after having opened thofe puftules, to cover the whole wound with linen dipped in lye, which was ordered to be repeated four times a day. The patient complaining of a tafte of *gas hydrogène fulfuré*, I prefcribed her about half a drachm of carbonate of pot-afh, or fixed vegetable alkali in a pint of water, fweetened with fome fyrup, and recommended her to drink freely. The following day a ftrong fmell of hepatic gas was perceived in the cloaths, and in the urine, which alfo fhewed on its furface, having ftood for fome time, an oily matter of the thicknefs of two lines, and with which the cloaths were likewife covered. The fixth day the wound began to dry, and on the tenth it was nearly healed; two purgatives terminated the cure, and the patient found herfelf quite recovered.

*Obferv.* 2. The 16th of Floreal, returning on horfeback from the country in exceedingly hot weather, a very heavy ftorm, attended with continual great flafhes of lightning and thunder, overtook me on the way. The horfe fuddenly fell down, and I felt that fame moment a great heat on my face and hands, and perceived that the hairs of my eye-brows and beard were burnt, and likewife the mane of my horfe, which died three days after. The firft twelve days after this accident, I felt nothing but fome heat over my whole body, attended with a profufe perfpiration; and as I happened to have a cold at the time, my expectoration went not only regularly on, but even with more facility. During this time I had a flight tafte of hepatic gas in my mouth, which fmell was alfo perceived by thofe who approached me. In this quiet ftate I almoft began to forget the accident; when, on the twelfth day, I was fuddenly feized with violent convulfions in my arms, which lafted an hour, and at the fame time I was attacked by a violent head-ach and great pains in the throat; the following night was very reftlefs, the head-ach more violent, and I was nearly fuffocated. I was immediately bled at the foot, which I repeated fome hours after, and by that means the head-ach diminifhed confiderably, my refpiration became more free, and I could fwallow better; but the hepatic hepatic fmell and tafte got almoft intolerable. I flept a few hours during the night, and when I awoke I obferved that my head and face began to fwell, which increafed every moment; befides this general fwelling, partial tumours rofe on my face as well as on my head, of which the fmalleft were of the fize of a nut, and at the top of the largeft, fmaller ones appeared; they were all hard and red, and caufed exceffive heat and pains. My head, whofe circumference in its natural ftate is about twenty-two inches, had now almoft increafed to above fifty; in proportion as it was fwelling, the head ach diminifhed, together with the pains in the throat; and when it had reached the higheft degree, it was entirely gone. My head, however, diminifhed by degrees, as it had begun to fwell, and on the 10th day the epidermis feparated, which was finifhed in a few days. My urine exhaled, like that of the woman, an infupportable hepatic fmell, and my abundant perfpirations had the fame fmell, which was fo ftrong, that it was almoft impoffible for any body to ftand near me; when my urine had ftood for a while, an oily cuticula, two lines thick, appeared upon it. The treatment I underwent was very fimple; after being twice bled, I kept a moft rigorous regimen; and the good effect I had obferved from alkali induced me to ufe it likewife; I took therefore fifteen drops of ammonia, or alkali volatile fluor every fix hours, and, indeed, at each dofe I perceived a confiderable relief. We prefent thefe obfervations for the public, to decide more juftly by further obfervations, what advantage may be derived from alkalis in curing accidents occafioned by lightning. *(Ibid.)*

---

*Extract of a Memoir on the Mildew, and the Acid contained therein; read at the Philomatic Society at Paris, by Cit.* CHANTRAN.

CIT. CHANTRAN having remarked that the ftalks bearing blighted ears do not differ from others; and that thefe ears are, at the fame time, compofed of good and bad grains, thinks it more likely that this difeafe of the corn does not pre-exift in the germ of the feeds, as has been commonly believed. By analyfing 46 grains of mildew, he found it to contain an acid eafy to be extracted by thofe means of the analyfis, which could not fufficiently act upon it as to produce and form it. Boiling water infufed with it, quickly reddens blue vegetable tinctures, whereas the refiduum of this infufion does not any more fhow that character. The mildew, freed from its acid, and calcined, at the accefs of air affords a fmell like burned horn, and the refiduum is fix times more than that of the fame quantity of meal treated in the fame way. This, with microfcopical obfervations, feems, according to Cit. Chantran, to prove the animality of that fubftance, and to mark a greater difference from the meal of corn, than can be produced by a fimple difeafe.

The acid of mildew is not volatile, and may be obtained concentrated by diftillation. It forms with lime and ammonia an in-

ſoluble ſalt. This laſt character diſtinguiſhes it from phoſphoric acid. Combined with potaſh, it affords a ſalt cryſtallized in ſmall needles, eaſily deliquating, and of a bitter taſte. The carbonate of lime is decompoſed by it.

---

Cit. Buniva has read a very intereſting Memoir, before the Society of Medicine at Paris, on the Epizootic Diſeaſe of Cats. Theſe animals are ſubject to chronic as well as to acute diſeaſes, epizootic (a word which anſwers to epidemic in man) malige. He deſcribes all the different ſymptoms which attended the acute diſeaſe, by which cats were lately attacked, and periſhed in great numbers, in Italy, France, and Germany; and of which the moſt remarkable is, that, after the ſecond day of the diſeaſe, they loſe the property of electric ſparks being drawn from their back, a phenomenon very obſervable in their ſound ſtate; and likewiſe, thrown from any height, they fall no more on their feet, which they commonly do. The diſſection of the body ſhows gangrenous ſpots in the bowels, &c. Cit. Buniva then enters upon an enquiry into Dr. Mitchell's theory, who derived the origin of contagion from the nitrous oxydated gas, or nitrous dephlogiſticated air, of Prieſtly (ſepton Mitch.); but he found this opinion did not agree with ſeveral experiments he purpoſely made with Vauquelin. This epizootic of cats communicates, according to his obſervations, to cattle, and vice verſa. It is likewiſe obſerved, that ſuch epizootics are fore-runners of an epidemic, and on that account they deſerve the attention of medical men. *(Reçueil Periodique, Vol. XLIV.)*

---

The Ruſſian Count Mussin-Puschkin has ſucceeded in rendering the metal of platina cryſtallizable, by treating it according to Richter's method, with vitriolated tartar or ſulphat of pot-aſh, and reducing it by means of common ſalt, inſtead of natron. This experiment, however, muſt be made with the greateſt exactneſs, and the fire ought not to be ſtronger than juſt to produce an uniform and ſhining cuticula in the middle of the crucible, becauſe if the fire is too vehement, the metallic particles will go to the bottom of the veſſel in ſmall maſſes, in form of trees, and the cryſtallization in vain be expected. The cryſtals cover the inferior page of the above cuticula in the ſhape of ſmall rhomboid priſms, plates, &c. and have at the firſt ſight, the appearance of the fineſt cryſtals of antimony. The Count gives a ſatisfactory explanation of this proceſs of cryſtallization.——A ſolution of platina in aqua regia, conſiſting of four parts of common ſalt, and five parts of nitrous acid diluted with three parts of water, made in the ſand bath, affords, when the retort is become cool, and two or three parts of the fluid are gone into the receiver, three ſorts of ſalt, cubic nitre, common ſalt faintly tainted with platina, and a new ſalt in form of quadrangular plates, lying in ſtrata one upon another, of a beautiful dark red colour, which particularly diſtinguiſhes itſelf by its eaſy ſolubility from all other ſalts of that metal. It has a gentle aſtringent taſte, and ſuffers eaſy reduction before the tube; but is afterwards not attracted by the loadſtone, nor malleable, except

 when when treated with nitric acid. It is probable, that on account of the quantity of water of cryſtallization which it contains, a greater coheſion of the particles of this metal is produced, than in any other ſalt of platina; a circumſtance, which will probably contribute much to its being with leſs difficulty worked by the hammer, particularly if the metal, after being reduced from the ſalt, is treated with nitric acid. *(Vide Crell's Chemical Annals, in German, No. 2, 1800.)*

---

The ſame gentleman relates ſome experiments reſpecting the decompoſition of red lead in nitric acid; according to which, this acid ſeems to have a conſiderable effect upon it, contrary to the experiments of Vauquelin. A ſolution of the red lead in that acid is produced in the ſand bath, which, after being ſome time expoſed to the action of light, lets fall cryſtals of a dark red colour, receiving by degrees a metallic appearance. If any acid is poured upon them, a part remains almoſt unaltered, while another forms a precipitation of a yellow colour; and a part of the chromium contained in the red lead ſeems by this to be changed into a calx.—It is likewiſe remarked, that the chromiate of ſilver is decompounded by muriatic acid; but the acid of chromium is with the greateſt difficulty changed into the ſtate of a metallic calx. After a part of the chromium calx is precipitated from the green ſolution of the chromiate of ſilver by alkalis, it produces, by being filtered, a yellowiſh colour; and metallic ſolutions in mineral acids, decompounded by it, afford always chromiates of metals.—The chromiate of iron, an ore which is found in Siberia, and which reſiſts the action of cauſtic alkali as well as nitre in the dry way, is, according to the experiments of the ſame gentleman, eaſily decompounded by detonating it with nitre; a proceſs, he thinks, by which metals moſt obſtinate to analyſe may be decompounded. *(Ibid. No. 3.)*

---

Prof. Tromsdorf, of Erfurt, has analiyed the blue chalcedony from Siberia, in which, however, he could not trace any metallic particles by repeated experiments, though it ſeemed obvious, from its dark colour, to contain them. The reſult of his experiments was, that it conſiſted of nothing but the pureſt ſiliceous earth; a circumſtance ſo much more remarkable, as other ſpecies of chalcedony have never been found, only pure ſiliceous earths, which appears from the experiments of different chemiſts; viz. Mr. *Bindheim, (Tranſactions of the Society of Friends of Natural Hiſtory, Vol. iii. p.* 429, *in German,)* found in the common chalcedony ſiliceous, aluminous, and calcareous earths, and iron; Mr. *Bergman (in his Opuscul. Chem. Vol. ii. p.* 60*)*; and Mr. *Fuchs (in Leny's Minerological Compendium, p.* 47, *in German),* beſides ſiliceous earth, aluminous and calcareous earths, and the calces of copper and iron. The blue colour of that foſſil ſeems therefore to depend on a particular ſtate of aggregation in its particles, by which a particular refraction is cauſed. Thus nature forms, in a way inconceivable to us, from from the ſame ſubſtance, ſiliceous earth, the blue chalcedony, the noble opal, the clear and tranſparent rock cryſtal, and the opaque flint ſtone. (*Ibid. No.* 3.)

---

Mr. Lowitz, of Peterſburgh, in the ſame Journal, has given a memoir on the nature of the common kali, imperfectly ſaturated with carbonic acid. It is known that we obtain the common kali by the combuſtion and calcination of vegetable bodies in a ſtate but imperfectly ſaturated with carbonic acid. This has hitherto been thought to be merely accidental, in the ſame manner as if, in the artificial compoſition of any neutral ſalt, either of its conſtituent parts is added in too great a proportion; which proceſs is diſtinguiſhed by the name of ſuperſaturation. Now, it may be conſidered either as mechanical or chemical; in the firſt caſe it is accidental, and the ſuper-abundant particle, whether an acid or alkali, eaſily and perfectly ſeparates by cryſtallization, during the drying of the cryſtals, and by alkohol, if the neutral ſalt is inſoluble in it. On the contrary, in the chemical ſuperſaturation, the ſeparation of the ſuper-abundant particle is by no means effected in this way, but a proper combination of a ſalt is formed, poſſeſſing properties different from thoſe, which are obvious in it when it is perfectly and equally ſaturated. The borax, ſublimate, &c. are inſtances of the kind. By different experiments relating to the above obſervation, the common alkali has proved to be alſo a ſalt, which, like borax, is chemically ſuperſaturated with its alkaline baſis. (*Ibid.*)

---

The ſame chemiſt attributes the purifying property of the charcoal to a chemical action, though it has been conſidered, by the moſt celebrated chemiſts, merely to act mechanically in rendering different fluids pure and clear. But if this only depended on its porous and ſpongy quality, it would not be poſſible for it to have any effect by being reduced to a powder; of which, however, experience ſhows the contrary, as it appears more efficacious the finer it is pulveriſed; and it keeps that property, even when by wetting it with water its poroſity is entirely deſtroyed. If the diſcolouring of any fluid by the powder of charcoal, depended merely upon its mechanically imbibing the colouring particles, it would be practicable to make them again appear by a proper treatment, after having ſeparated the powder from the diſcoloured fluid. But this is by no means to be effected; likewiſe the ſmell, or oily particles which have been taken from any fluid by coals, would be perceived in them afterwards, which is never obſerved. The powder of the charcoal, beſides, is able to diſcolour a chemical ſolution of colouring matter in water or ſpirits; and this muſt therefore probably be effected chemically. It appears by this, that the powder of charcoal enters into a chemical combination with thoſe particles, from which its addition frees different fluids. The chemical affinity of them ſeems ſtill more evident by the following experiment. One or two drachms of the powder of charcoal being, ing, by washing, perfectly freed from any adhering alkaline particles, are boiled in a considerable quantity of water till all the air contained in its pores is expelled, and itself sinks to the bottom from its greater specific gravity. Having distributed this into four different glasses, and added to the first a portion of any gross oil; to the second, some vitriolic ether; to the third, alkohol, all of which are lighter than water, the powder of the charcoal combines itself with these substances, and swims with them upon the surface of the water; but if the essential oil of cloves (oleum caryophyllorum) or any other ponderous oil, is put into the fourth glass, the powder of the charcoal will sink with it to the bottom, and the water become quite clear; all which facts are difficult to be explained in a mechanical manner; it can only be allowed, that the powder of charcoal purifies turbid fluids by a mechanic action. *(Ibid. No. 3.)*

---

Mr. Lichtenberg, of Berlin, has found by his experiments, that a method of purifying nitric acid from sulphuric and muriatic acid by means of lead, as proposed by Vaquelin, did not answer the purpose. Sulphuric acid and nitric acid, which contained muriatic acid, were poured into a retort, in which a proportionable quantity of litharge of silver had been previously placed. Having distilled the acid, it was found to be still more impregnated with muriatic acid than before; the residuum in the retort consisted chiefly of nitrate of lead and a small quantity of sulphat of lead. The nitric acid is, indeed, freed by this method from sulphuric acid, but by no means from the muriatic acid. A more certain method of purifying nitric acid seems to be the following; namely, to dissolve the nitre in water previously to the distillation of the acid, and to purify it by the addition of the nitrate of silver, after which the nitre must be again crystallized before it is employed in the distillation.

---

Prof. Hermbstaedt has made several chemical experiments with the leaves and roots of the tulip tree, (Liriodendron tulipifera, L.) recommended as an useful bitter. The leaves of this tree, which possess somewhat of a sharp and bitter taste, were squeezed in a stone mortar, and the juice expressed; from which, after six hours, a glutinous matter of a green colour separated, coagulating by alkohol in the temperature of boiling water, and burning with the smell of horn, which, consequently, was occasioned by its vegetable albuminous matter. The juice being filtered and mixed with alkohol, still produced a portion of albuminous matter; the rest of it, treated accordingly, contained a great deal of saponaceous principle, some gum, a little resin, and likewise an astringent quality. Another portion of the juice, after being freed from albuminous matter by being twice boiled, was thickened to an extract of a very bitter taste. A similar extract was obtained from the juice of leaves which were repeatedly boiled in water. Alkohol, digested with the leaves, extracts a tincture containing containing ſcarcely any thing but ſaponaceous matter. The rinds of the younger roots have a ſmell and taſte much like Canella Alba; and this aromatic matter is extracted by alkohol, together with ſome reſin and eſſential oil.

---

We are in poſſeſſion of a Memoir of the ſame able Chemiſt, on the chemical analyſis of animal bodies, ſimilar to what has been publiſhed by him on the vegetable productions, which we ſhall take an early opportunity of communicating to our readers.

---

We find the uſe of the Semina Phellandrii Aquatici, L. which have been mentioned in this Journal as a remedy in pulmonary conſumptions, likewiſe recommended by a phyſician in Saxony in old ſore legs; and a caſe is related, where a patient who had had ſore legs many years, and which reſiſted all kinds of treatment, was at length cured in the courſe of ſeven weeks by Semina Phellandrii Aquatici, given in the doſe of one ounce per day.

---

Mr. Hirsh, a German dentiſt, recommends in the tooth-ach, grubs of Cynips Roſae, an inſect which cauſes the excreſcences found on wild roſe buſhes, and known under the name of Spongia Cynoſbatos, or Fungus Bedeguar, in which thoſe little worms are met with. They are to be ſqueezed between the fingers, and the affected tooth or gums repeatedly rubbed with them; when the tooth-ach ceaſes, or, at leaſt, is much diminiſhed. *(Hufeland's Practical Journal.)*

---

Brugnatelli propoſes a new arrangement of mineral waters, by dividing them into four claſſes:

1. Saline mineral waters, mineraliſed by terreous, alkaline, or metallic ſalts.

2. Saline acidulated mineral waters, which contain a free acid beſides the ſalts.

3. Saline ſulphureous mineral waters, in which, beſides the ſalts, ſulphur and ſimilar parts are met with.

4. Sulphureous mineral waters, which contain ſulphur in different proportions and ſtates, but never any ſalt. *(Annali di Chemica et Storia Naturale, Tom. XVII.* 1798, No. 101.*)*

---

The ſame chemiſt conſiders the formation of ethers as an oxygenation, or, as he calls it, thermoxygenation, of alkohol with an oxyd gas, gas oxyde. *(Ibid.)*

---

The Obſidian, a volcanic foſſil of Iceland, has been lately analyſed by Prof. Tromſdorf, of Erfurt, and found to contain 63, ſiliceous earth, 20,5 aluminous, and 13,5 oxyd of iron. A black feldſpar, found in the Baſalt, contains, according to the ſame, 66,5 aluminous earth, 15,0 ſiliceous, 6,5 oxyd of iron, and 9,0 oxyd of manganeſe. *(Annales de Chemie, No.* 101.)

---

Prof.

